# Unilateral Resistance Training Strategies for Boosting Rehabilitation: An Expert Survey

**DOI:** 10.3390/jfmk10040425

**Published:** 2025-11-02

**Authors:** Ioana Mădălina Petre, Mircea Boșcoianu, Petronela Mocanu

**Affiliations:** 1 Department of Industrial Engineering and Management, Transilvania University of Brașov, 500036 Brașov, Romania; boscoianu.mircea@yahoo.com; 2 Department of Motric Performance, Transilvania University of Brașov, 500036 Brașov, Romania; petronela.mocanu@unitbv.ro

**Keywords:** rehabilitation, resistance training, unilateral resistance training exercises, sports rehabilitation, sports training, dedicated rehabilitation strategies

## Abstract

**Objectives**: This research paper proposes an innovative framework for developing adaptive and dedicated rehabilitation strategies based on the perceptions of specialists in sports rehabilitation (RT), sports training (AR) and with mixed expertise (RT+AR) regarding advanced resistance training methods, including Effort-Based Training (EBT-3/7), Cluster Training (CT), Rest-Pause Training (RPT) and Post-Activation Potentiation (PAP). The aim of this paper was to develop a tailored strategy for rehabilitation programs, grounded in a targeted selection of training methods, short-term periodization and exercises structured around key training variables such as frequency, intensity and volume. **Methods**: In order to reach this objective, a quantitative research method is proposed, aiming to identify the experts’ opinion on the way of managing and integrating Unilateral Resistance Training Exercise (URTE). Data processing and analysis were conducted by means of specific tests supplied by the SPSS Statistics for Windows (version 20.0, IBM Corp., Armonk, NY, USA). **Results**: The findings indicate that EBT-3/7 is perceived as the most effective method for rehabilitation with minimal injury risk, whereas CT and PAP are associated with performance benefits but higher perceived injury risk. RT+AR specialists reported more frequent use of these methods and higher perceived effectiveness. Additionally, they demonstrated superior operational and dynamic capabilities compared to single-domain specialists. **Conclusions**: According to specialists’ opinions, URTE is effective for post-injury rehabilitation, with combined rehabilitation and training expertise enhancing utilization, perceived effectiveness and implementation of personalized, performance-oriented strategies.

## 1. Introduction

One of the primary challenges faced by practitioners in resistance training is determining how to optimally select exercises to maximize training adaptations and performance outcomes [1]. In general, weight room exercises can be classified as either unilateral or bilateral, and the choice between these movement patterns can significantly influence strength development, neuromuscular coordination and injury risk, making exercise selection a critical component in designing resistance training programs intended to maximize muscular adaptations [2].

Mullican et al. suggested that unilateral training may promote more favorable strength adaptations, potentially leading to greater strength gains in the trained limb while maintaining comparable improvements in bilateral strength relative to conventional bilateral training [3]. According to Zhang et al., the effectiveness of training depends on the movement pattern: unilateral training enhances performance in unilateral power tasks, whereas bilateral training produces greater improvements in bilateral power tasks [4]. Moran et al. reported that both bilateral and unilateral resistance training are effective in enhancing horizontal movement performance, such as sprinting [5]. Kassiano et al. reported that while muscle hypertrophy showed no significant difference between bilateral and unilateral training, strength adaptations appeared to be task-specific [6]. Unilateral training is often promoted for its functional advantages and adherence to the principle of specificity due to the bilateral deficit (BLD)—a neuromuscular phenomenon where combined bilateral force is less than the sum of unilateral outputs—yet evidence shows that the presence and magnitude of the BLD vary widely across individuals, with factors such as training status, task familiarity, and neuromuscular control determining whether a deficit, no effect, or even bilateral facilitation is observed [7].

Recent research highlights the benefits of incorporating Unilateral Resistance Training Exercise (URTE) into rehabilitation and athletic programs. URTE not only addresses limb imbalances and asymmetries resulting from injury but also enhances neuromuscular control, muscle hypertrophy and functional performance. By focusing on one limb at a time, URTE facilitates targeted strength gains, improved motor unit recruitment and greater neural drive, which are essential for both rehabilitation and performance optimization. Studies have demonstrated that unilateral exercises, such as split squats, single-leg presses and alternate-leg bounds, can improve strength, jump performance, sprinting ability and change-of-direction speed, often producing comparable or even superior outcomes relative to traditional bilateral movements [1,4,7].

Moreover, unilateral training has been shown to induce cross-education effects, where training the non-dominant or uninjured limb results in strength and balance improvements in the contralateral limb. The study carried out by Razian et al. found that unilateral training of the non-dominant leg led to greater cross-education effects, improving strength and balance in the contralateral limb [8]. This approach may be particularly useful in rehabilitation when one limb is injured or immobilized [8], allowing athletes to maintain neuromuscular function and mitigate detraining effects in the affected limb.

Additionally, recent systematic reviews and meta-analyses indicate that unilateral training promotes significant neuromuscular adaptations, including increased muscle thickness, fiber-type shifts, enhanced neural activation and improved rate of force development [9,10,11,12,13]. These adaptations collectively support maximal strength development, functional recovery and performance enhancement, making URTE a valuable tool in short-term training blocks of 4–6 weeks, during which high-performance athletes can actively rebuild strength, confidence and motor control following injury. Integrating unilateral exercises within a periodized program allows practitioners to strategically manage training loads while promoting balanced strength development and reducing the risk of re-injury, ultimately contributing to more efficient and effective rehabilitation outcomes.

What makes this approach unique is the emphasis on intensifying effort—not only through the regulation of key training parameters such as frequency, intensity and volume—but also by incorporating specific resistance training techniques tailored to the individual. In this context, the main intensification strategies highlighted in specialized literature that could contribute to the acceleration of athletes’ rehabilitation are presented in Table 1.

These strategies are essential for optimizing the effectiveness of URTE protocols during both rehabilitation and performance restoration phases. The first two strategies—progressive overload and volume progression—are successfully implemented by the athletes in their training, often integrating periodization elements such as planned variations in intensity, volume and exercise selection to maximize long-term performance. Progressive overload, through the gradual increase in resistance or exercise complexity, ensures that neuromuscular and musculoskeletal systems are continually challenged without exceeding tissue tolerance, allowing safe and effective adaptation. Volume progression, achieved by incrementally increasing repetitions, sets or total work, complements progressive overload by providing sufficient training stimulus for hypertrophy, strength and endurance gains. Rehabilitation programs should introduce load and resistance gradually, progressively increasing intensity as the athlete’s tissue tolerance and functional capacity improve, aligning with periodization principles [16].

The third strategy focuses on accelerating performance gains by refining the application of progressive overload and volume manipulation. While it is conceptually an extension of the first two strategies, it emphasizes strategically pushing the athlete closer to their maximal capacity in later rehabilitation phases, carefully balancing intensity with safety considerations. RIR can be incorporated in later rehabilitation stages by instructing athletes to stop 1–2 repetitions short of failure, while early phases maintain lower intensities to protect healing tissues [14]. Although rarely discussed in the literature, this approach can expedite strength and power development, particularly for athletes aiming to return to high-level sport-specific demands in the shortest safe timeframe.

The fourth strategy involves the adaptive manipulation of total set impulse, defined by both the duration and intensity of each set. By adjusting contraction types, tempo, range of motion and time under tension, this strategy provides innovative solutions to tailor the training stimulus to the athlete’s current capacity and stage of rehabilitation. For example, Petre et al. analyzed the characteristics of unique sets in a maximum repetitions protocol, demonstrating how variations in tempo, contraction type and set duration influence muscular response [17].

One of the main gaps in current research on unilateral training strategies in rehabilitation is the lack of a clear consensus on the most effective techniques and implementation protocols. While evidence shows that unilateral training can improve strength and functional outcomes in affected limbs, few studies have systematically examined the impact of different training intensification methods—such as repetition style modifications, volume progression or reductions in repetitions in reserve—on rehabilitation outcomes. Moreover, most existing research focuses primarily on the physiological effects of unilateral training, without integrating the perspectives of clinical and sports training experts. This lack of integration between scientific research and practical expertise may limit the applicability and effectiveness of unilateral training strategies in rehabilitation.

Therefore, the present study aims to address this research gap by collecting and analyzing expert opinions from rehabilitation and sports performance professionals. Through a structured survey, the study explores their perceptions and recommendations regarding the most effective unilateral training strategies, taking into account rehabilitation stages and individual patient characteristics.

The study provides a novel perspective by simultaneously assessing the perceptions of rehabilitation and training specialists regarding multiple advanced resistance training methods in high-performance contexts. The research highlights the enhanced effectiveness, professional engagement and dynamic capabilities of specialists combining both rehabilitation and training expertise. Moreover, it identifies the practical trade-offs between rehabilitation efficacy and perceived injury risk across different methods, offering evidence-based guidance for integrating advanced training strategies into individualized post-injury rehabilitation programs.

The expected findings will contribute to the development of more scientifically grounded and clinically relevant training protocols, facilitating the more effective integration of unilateral exercises into rehabilitation programs.

## 2. Materials and Methods

### 2.1. Unilateral Resistance Training Exercises

Within URTE, the methodological emphasis is placed on balancing core training variables—effort, volume and load—to optimize neuromuscular recovery in injured athletes. Unlike traditional bilateral protocols, URTE allows for localized stimulation of the unaffected limb while eliciting cross-education effects that benefit the injured side [18]. This approach leverages the body’s ability to generate neural adaptations through unilateral loading, thereby preserving strength and function during periods of immobilization or asymmetrical training capacity [19].

Adjustments to effort and training volume are made progressively, ensuring that the athlete receives sufficient stimulus to promote adaptation without overloading compromised structures. The integration of variable-intensity schemes, including eccentric loading and set-extending methods, further enhances motor unit recruitment and neuromuscular control [20]. These strategies are particularly effective in early-to-mid stages of rehabilitation, supporting a gradual return to bilateral performance while minimizing re-injury risk.

(a) Effort-based training 3/7 (EBT-3/7)

The reference-based method is Effort-based training equipped with Legeard-Laurent innovative version or the 3/7 Strategy (EBT-3/7). In the case of conventional Effort-Based Training, the core parameters of a training session aim to harmonize volume, frequency and intensity to enhance overall effectiveness. For example, a typical protocol may involve: 1–2 maximal effort sets, 5–10 repetitions per set, 1–4 exercises per muscle group, and 5–6 exercises per training session. In contrast, the EBT-3/7 version takes training efficiency to a higher level by combining two key effects: mechanical stress, achieved through relatively heavy loads and metabolic stress, induced through shorter rest periods. This hybrid approach amplifies training stimuli and adaptation.

The inspiration for the EBT-3/7 method comes from the mechanisms specific to Blood Flow Restriction Training (BFRT), which also combines high metabolic demand with lower overall volume to promote muscular adaptations effectively [21,22]. Research on athletes indicates that combining BFRT with moderate-intensity interval training enhances aerobic and anaerobic capacities, as well as lower extremity performance [23]. A systematic review and meta-analysis found that BFRT significantly improves muscle strength and hypertrophy in athletes, suggesting its effectiveness as an innovative training modality [24].

In the 3/7 method, a typical load is set at approximately 70% of one-repetition maximum (1RM), which traditionally allows for 10–12 repetitions in a conventional set. These values are similar to the case of unilateral exercises, with the mention that even better results are achieved by benefiting from increased control of movement. However, this method modifies the approach by implementing the Legeard-Laurent strategy, consisting of three sets per exercise with rest intervals of 150–180 s between sets. Each set follows a specific configuration: 3 repetitions, 15 s rest, 4 repetitions, 15 s rest, 5 repetitions, 15 s rest, 6 repetitions, 15 s rest, 7 repetitions [25]. The study compared two training protocols: the 3/7 method, which consists of 5 sets with an increasing number of repetitions (3–7) and short rest intervals (15 s), and the 8 × 6 method, with 8 sets of 6 repetitions and 150 s rest intervals. Results showed that the 3/7 method led to significantly greater improvements in 1RM load and maximal voluntary isometric contraction, as well as a greater increase in biceps brachii thickness, compared to the 8 × 6 method.

However, the method has not been tested for unilateral exercises, leaving a gap in the literature regarding its applicability and effectiveness in single-limb training. Given that unilateral exercises allow for greater focus on movement control, neuromuscular activation, and the correction of limb imbalances, applying the 3/7 method in this context could potentially enhance strength gains, hypertrophy and functional performance more effectively than in bilateral settings. Investigating the EBT 3/7 protocol with unilateral movements would therefore provide valuable insights for optimizing rehabilitation and performance programs, particularly for athletes recovering from limb-specific injuries or aiming to correct asymmetries.

(b) Cluster Training (CT): cluster-sets and drop-sets

Set extenders represent another innovation for enhancing the quality of training sets inspired by the concepts of total impulse per set, in which the basic parameters are the instantaneous force and the duration of the sets, without strictly taking into account the number of repetitions per set and the total number of repetitions. Instead of the classic 5 sets × 5 reps = 25 total repetitions, typically performed at 65–75% of one-repetition maximum (1RM), CT modifies the structure by inserting approximately 20 s of rest between each repetition. As a result, the set becomes 1 rep + 20 s rest + 1 rep + 20 s rest, and so on.

This configuration allows the use of significantly heavier loads, up to 85% of 1RM, effectively intensifying the training stimulus [26]. Short intra-set rest intervals reduce neuromuscular fatigue, enabling better maintenance of bar speed and power output, particularly during high-load sessions [27].

Cluster training (CT) was shown to produce similar improvements in muscle strength and power compared to traditional set structures, consistent with findings that intra-set rest periods allow maintenance of load and performance without additional gains in muscular outcomes [26]. Moreover, cluster sets have been shown to elicit less perceived exertion while maintaining similar or superior training outcomes compared to traditional straight sets. This is supported by Cui et al., who concluded that cluster training effectively mitigates exercise-induced fatigue and enhances maximum strength within an 8-week period, making it especially useful during shorter training phases [28]. Cluster sets could be readily integrated into unilateral resistance training sessions, providing the advantage of high-density training—a critical factor for performance athletes—while maintaining good control over injury risk.

Drop sets are another set-extending strategy that increases metabolic stress and muscle fiber recruitment by reducing the load after reaching momentary failure. Acute studies have shown that drop sets can elevate muscle activation and fatigue more rapidly than traditional methods.

Drop sets are another set-extending strategy that increases metabolic stress and muscle fiber recruitment by reducing the load after reaching momentary failure. Acute studies have shown that drop sets can elevate muscle activation and fatigue more rapidly than traditional methods [29].

(c) Rest-pause training (RPT)

The configuration of a set in RPT extends the classic set by inserting short rest periods between clusters of repetitions. A typical RPT set structure might look like 5 reps + 20 s rest + 4 reps + 20 s rest + …

This method allows lifters to perform a higher total volume with heavier loads by partially recovering between mini-sets, thus delaying fatigue accumulation [30]. RPT has been shown to effectively enhance muscle strength and hypertrophy by allowing continuation of high-load sets after brief intra-set rest intervals, thereby combining mechanical tension with increased metabolic stress [31].

Recent research provides further evidence that RPT significantly increases levels of insulin-like growth factor 1 and the follistatin/myostatin ratio compared to traditional resistance training, indicating enhanced muscle protein synthesis and hypertrophic potential [31]. Studies suggest that RPT can enhance strength and muscle activation compared to traditional sets by allowing lifters to partially recover between mini-sets, maintaining higher intensity and total training volume throughout the extended set [30,32].

(d) Post-activation potential (PAP)

PAP is an effective strategy for enhancing explosive performance and neuromuscular coordination, as demonstrated in well-trained athletes during activities such as jumping and throwing [33,34]. Additionally, PAP protocols can effectively enhance jump performance in athletic populations, although outcomes vary based on protocol specifics and individual differences [35]. Recent findings further suggest that torque regulation and neuromuscular control are strongly influenced by the nature of contraction and feedback conditions [36], indicating that PAP-induced benefits may depend not only on load and timing but also on how contraction strategies are structured and monitored.

The findings of Del Vecchio et al. highlight that motor unit synchronization is driven by multiple independent sources of common synaptic input, emphasizing the complexity of neuromuscular coordination [37]. This aligns with the rationale for using PAP, which has been shown to acutely enhance motor unit recruitment and synchronization, thereby improving explosive power and neuromuscular efficiency.

In practice, PAP can be implemented through supersets designed to stimulate both ends of the Force–Speed curve, thereby optimizing total momentum during a training set. Athletes benefit from this approach because it develops both maximal strength (force) and explosive power (force × speed, with an emphasis on velocity) within the same session. The Force–Speed curve illustrates that higher force typically occurs at lower velocity, whereas higher velocity requires less force, with the optimal training range often located between 40 and 60% of 1RM depending on the exercise. By pairing contrasting exercises in a superset, athletes can train these complementary qualities more effectively, maximizing strength, power and neuromuscular adaptation.

### 2.2. Research Method and Representative Sample

The study employed a structured questionnaire consisting of seven sections designed to assess specialists’ perceptions of advanced training methods in the rehabilitation of high-performance athletes. First section collected demographic and professional background information, including field of activity, years of experience and educational level. The next four sections examined the use of specific methods—EBT-3/7, CT, RPT and PAP. For each method, respondents were asked to report frequency of application, perceived advantages and disadvantages, overall effectiveness in rehabilitation and relevant efficiency indicators such as recovery, muscular performance and neuromuscular adaptation. Next section provided an overall evaluation of the methods, asking participants to identify the most effective technique for rehabilitation and the one associated with the greatest risk of injury. The final section of the questionnaire assessed broader operational and dynamic capabilities in rehabilitation and training, including the acquisition, integration and application of knowledge, as well as adaptive, absorptive and innovative capacities.

To assess reliability, Cronbach’s alpha was calculated, obtaining a value of 0.861, which suggests good internal consistency among the questions.

G*Power software (version 3.1.9.4, University of Düsseldorf, Düsseldorf, Germany) was used to calculate the sample size, considering a small effect size of 0.55 for an untrained or recreationally trained population [38], power (1–β) of 0.8 and an α level of 0.05. Based on these parameters, a minimum of 16 participants per group was determined as the required sample size. For the purposes of this study, three equal samples of 20 participants were recruited, comprising sports rehabilitation specialists (RT), sports training specialists (AR) and specialists practicing in both areas (RT+AR). This grouping allowed for a comprehensive comparison of perceptions regarding advanced training methods in the rehabilitation of high-performance athletes. However, some participants withdrew or did not complete the intervention; thus, only fully completed questionnaires were analyzed. To ensure that the groups remained comparable, the final analysis included 16 participants per group. Inclusion criteria for this study were as follows: (i) specialists currently working in sports rehabilitation, sports training or both; (ii) at least one year of professional experience; and (iii) relevant educational background in physiotherapy, kinesiology, sports science, or equivalent certifications. After excluding inactive or unqualified respondents, a total of 45 fully completed questionnaires were retained for analysis.

The subjects were informed of the research procedure and provided their consent. The study was conducted in accordance with the Declaration of Helsinki; responses were collected anonymously and analyzed to identify both quantitative trends and qualitative insights regarding the integration of the methods into rehabilitation practice.

### 2.3. Data Analysis

Data were processed and analyzed using SPSS Statistics for Windows (version 20.0, IBM Corp., Armonk, NY, USA). Analyses included frequency distributions, descriptive statistics, one-way ANOVA and corresponding post hoc tests. The reliability of the questionnaire was evaluated using Cronbach’s alpha. Tukey’s HSD test was applied when the assumption of equal variances was met, whereas the Games–Howell test was used in cases of unequal variances. Statistical significance was set at *p* < 0.05 for all analyses.

We hypothesized that:

**H1:**  *Combined specialists (RT+AR) report more frequent use of advanced resistance training methods compared to rehabilitation-only (RT) or training-only (AR) specialists*.

**H2:**  *The perception of the training methods’ effectiveness differs depending on the professional field of the specialists*.

**H2a:**  *EBT-3/7 is expected to be perceived as more effective for recovery and adaptation processes in rehabilitation by the combined RT+AR specialists*.

**H2b:**  *CT is hypothesized to have a positive impact on rehabilitation outcomes by enhancing muscle strength, neuromuscular adaptation and recovery capacity between sets*.

**H2c:**  *RPT is assumed to improve strength training performance while minimizing the risk of overtraining, thereby offering a safe and efficient method for progressive workload management in rehabilitation and athletic settings*.

**H2d:**  *PAP is expected to enhance explosive power and neuromuscular coordination, contributing to improved rehabilitation and performance outcomes*.

**H3:**  *Methods rated as more effective for rehabilitation are also associated with lower perceived injury risk, while methods perceived as riskier are less used in rehabilitation*.

**H4:**  *RT+AR specialists demonstrate higher operational and dynamic capabilities compared to single-domain specialists*.

## 3. Results

A total of 48 specialists participated in the study, divided equally into three groups: RT, AR and both areas. Regarding professional experience, 6 participants (12.5%) reported 1–3 years of experience, 16 participants (33.3%) 4–7 years, 13 participants (27.1%) 8–10 years and 13 participants (27.1%) had over 10 years of experience.

Regarding educational level, 37.5% of participants had a faculty degree, 52.1% held a master’s degree and 10.4% had a PhD. Out of 48 respondents, 22.9% are female and 77.1% are male, with ages ranging from 34 to 52 years (M = 43.9, SD = 4.93).

### 3.1. Effort-Based Training 3/7 (EBT-3/7)

In terms of practical use, more than half of the specialists (52.1%) reported using the EBT-3/7 method occasionally, while 35.4% indicated frequent use and 12.5% stated that they had never applied it. The overall mean score of utilization was 2.23 (SD = 0.66), reflecting a predominantly occasional practice.

Regarding the perceived advantages of the EBT-3/7 method, most specialists (43.8%) identified the optimization of muscle damage and protein synthesis as the main benefit. Quick learning of an effective training mode was reported by 25% of participants, while 12.5% emphasized its role in psychological awareness of set efficiency and in post-injury recovery. Only 6.3% mentioned the possibility of evaluating Reps in Reserve. With regard to disadvantages, the majority of specialists (56.3%) highlighted the low training volume as the main limitation of the EBT-3/7 method. Additionally, 31.3% reported a possible lack of motivation due to the reduced volume, while 12.5% mentioned difficulties in psychologically managing the end of sets.

In order to find certain differences among specialists regarding EBT-3/7 method, ANOVA analysis was performed. The results are presented in Table 2. When the ANOVA test is significant (*p* < 0.05), post hoc comparisons are performed. If Levene’s test indicates unequal variances (Levene Sig. < 0.05), the Games–Howell test is applied; otherwise, the Tukey test is used.

Analysis of EBT-3/7 perceptions revealed significant group differences in some areas. Utilization was higher in RT+AR (M = 2.56, SD = 0.51) compared to RT (M = 2.13, SD = 0.71) and AR (M = 2.00, SD = 063), *p* = 0.03. Regarding perceived effectiveness, all groups rated EBT-3/7 highly (M = 4.60, SD = 0.67), with no significant differences between them (*p* = 0.56). However, significant variation appeared in post-workout recovery scores (*p* < 0.05), where RT gave higher ratings (M = 4.69, SD = 0.60) than AR (M = 3.81, SD = 1.10). For muscular performance improvement (M = 4.63, SD = 0.60), injury risk reduction (M = 4.65, SD = 0.52) and readaptation of specific effort during post-injury recovery (M = 4.63, SD = 0.53), ratings were consistently high across all groups, with no significant differences (*p* > 0.21). Post hoc tests were applied to EBT3_7_utilization and EBT3_7_postworkout_recovery, as the ANOVA yielded *p*-values lower than 0.05 for these variables.

For EBT3_7_utilization Tukey HSD was applied (*p* = 0.44), which indicated a significant difference between AR and RT+AR, with higher utilization reported in the RT+AR group (M difference = 0.56, *p* = 0.03). No other pairwise comparisons reached statistical significance (all *p* > 0.10).

Post hoc Games–Howell comparisons revealed that RT rated post-workout recovery significantly higher than AR (M difference = 0.87, *p* = 0.02).

### 3.2. Cluster-Training: Cluster-Sets and Drop-Sets

Most specialists reported applying the cluster-sets method occasionally (66.7%), with 25% using it frequently and 8.3% never having applied it, indicating that this method is generally practiced, predominantly on an occasional basis. Similarly, the drop-sets method was used occasionally by the majority of specialists (64.6%), frequently by 31.3%, and never by 4.2%, suggesting it is also widely implemented with occasional use being most common. Regarding short breaks between repetitions in CT, 62.5% of specialists indicated that they help manage fatigue and increase efficiency, 33.3% reported that they allow for increased intensity without compromising performance and 42% considered that short breaks have no significant impact on performance.

The vast majority of specialists (91.7%) reported that CT has a positive impact on rehabilitation, helping muscle regeneration and improving performance, while 8.3% indicated that it has no significant effect.

The results obtained by ANOVA analysis are presented in Table 3.

For cluster sets and drop sets utilization, RT+AR reported highest score. Significant differences were observed only for cluster sets (*p* = 0.01). For perceived effectiveness in improving muscle strength, AR reported the highest scores (M = 4.81, SD = 0.40), followed by RT+AR (M = 4.31, SD = 0.60) and RT (M = 4.19, SD = 0.83), with a statistically significant difference among groups (*p* = 0.02). Strength improvement was rated positively across all groups (M = 4.75, SD = 0.43) and also recovery between sets (M = 4.54, SD = 0.58) with no significant differences between professional categories (*p* > 0.05). Neuromuscular adaptation received the highest overall ratings (M = 4.73, SD = 0.44), with RT+AR specialists reporting the highest scores (M = 4.94, SD = 0.25) and no significant differences among groups (*p* > 0.05).

Tukey post hoc comparisons revealed that for cluster sets utilization RT+AR showed significand difference compared to AR (M difference = 0.56, *p* = 0.01). Also, for effectiveness in improving muscle strength, Games Howell post hoc test showed that AR rated the method significantly higher than both RT (M difference = 0.62, *p* = 0.03) and RT+AR (M difference = 0.5, *p* = 0.02).

### 3.3. Rest-Pause Training (RPT)

Most specialists reported using the RPT method occasionally (72.9%), while 22.9% used it frequently and 4.2% had never applied it. Regarding the impact of short breaks, the vast majority (93.8%) indicated that they allow for increased workload and improved performance, 4.2% considered that they could lead to overtraining and excessive fatigue and 2.1% believed they provide no significant benefits compared to traditional sets.

In terms of perceived rehabilitation benefits, 43.8% of specialists indicated that RPT improves muscular endurance, 33.3% highlighted increased strength and muscle mass and 22.9% emphasized creating a balance between volume and intensity.

One-way ANOVA was conducted to examine differences across the three experimental groups (RT, AR and RT+AR). The obtained results are presented in Table 4.

The results indicated that, for RPT utilization, RT+AR (M = 1.38, SD = 0.5) showed significantly higher values than the other groups (*p* = 0.03). Tukey post hoc test indicated a significant difference between AR+RT and AR (M difference = 0.43, *p* = 0.02).

For increased workload, RT reported the highest mean score (M = 4.31, SD = 0.6). ANOVA revealed a significant difference between groups (*p* = 0.02). Post hoc Games–Howell tests revealed that for increased workload, RT rated the effect significantly higher than both RT+AR (M difference = 0.81, *p* = 0.04) and AR (M difference = 0.81, *p* = 0.04).

For muscle tissue rehabilitation, ratings were relatively consistent across groups (RT: M = 4.56, SD = 0.51; AR: M = 4.44, SD = 0.72; RT+AR: M = 4.75, SD = 0.44), with no significant differences observed (*p* = 0.31), indicating similar perceptions across professional domains.

Regarding neuromuscular response, RT+AR reported the highest ratings (M = 4.88, SD = 0.34) compared to AR (M = 4.44, SD = 0.62) and RT (M = 3.81, SD = 1.16), with the differences being statistically significant (*p* = 0.002). Post hoc Games–Howell tests revealed that RT+AR reported significantly higher effectiveness compared to RT (M difference = 1.06, *p* = 0.001).

Neuromuscular control was rated similarly across groups (RT: M = 4.31, SD = 0.87; AR: M = 4.56, SD = 0.51; RT+AR: M = 4.81, SD = 0.54) and no significant differences were found (*p* = 0.1).

### 3.4. Post-Activation Potential (PAP)

Among the 45 specialists surveyed, the use of PAP was reported as occasional by the majority (47.9%), with 33.3% using it frequently and 18.8% never applying the method. Regarding its perceived effects on sports performance, specialists were evenly split: half indicated that PAP helps improve neuromuscular coordination, while the other half believed that it increases strength and speed.

In terms of rehabilitation practices, most specialists (75%) reported implementing PAP by combining low-intensity exercises with high-velocity exercises, while 18.8% used a progression from bilateral to unilateral exercises. A small proportion (6.3%) indicated that they would not implement PAP in rehabilitation settings.

The results obtained by ANOVA analysis are presented in Table 5.

One way ANOVA analysis revealed some differences in PAP utilization across the three groups (*p ≤* 0.001). The highest mean value was observed for RT+AR (M = 1.5, SD = 0.51), followed by AR (M = 1.38, SD = 0.61) and RT (M = 0.56, SD = 0.62). Tukey post hoc test showed a significant difference between AR+RT and RT (M difference = 0.93, *p ≤* 0.001), and AR to RT (M differences = 0.81, *p* = 0.001).

Specialists rated the effectiveness of PAP for improving explosive power, reaction speed and neuromuscular coordination. For explosive power, RT+AR reported the highest mean rating (M = 4.88, SD = 0.5), followed by RT (M = 4.25, SD = 0.77) and AR (M = 3.81, SD = 1.16). ANOVA revealed a significant difference between groups for explosive power (*p* = 0.004). Games–Howell post hoc test revealed significant differences between RT+AR and both RT (M difference = 0.62, *p* = 0.03) and AR (M difference = 1.06, *p* = 0.008). This indicates that RT+AR perceive PAP as significantly more effective for enhancing explosive power compared to those working in only one domain. No significant difference was observed between the RT and AR.

For reaction speed, mean ratings were 4.81 (SD = 0.4) for RT+AR, 4.38 (SD = 1.08) for RT and 4.06 (SD = 1.43) for AR. ANOVA showed no significant differences between groups (*p* = 0.14), suggesting similar perceptions across all professional categories.

Regarding neuromuscular coordination, specialists rated PAP very highly, with RT+AR giving a perfect mean score, followed by AR (M = 4.81, SD = 0.54) and RT (M = 4.44, SD = 0.89). Differences between groups were statistically significant (*p* = 0.03), so, Games Howell post hoc test was performed. Significant differences were identified between RT+AR and RT (M difference = 0.56, *p* = 0.05).

### 3.5. General Evaluation

Most specialists rated EBT as the most effective method for rehabilitation, with 54.2% selecting it, while RPT was also frequently chosen (39.6%). CT was considered the most effective by 4.2% of respondents and PAP by 2.1%.

When asked about methods presenting the highest risk of injury, CT was most frequently selected (47.9%), followed by PAP (39.6%) and RPT (12.5%).

This suggests a trade-off in specialists’ perceptions: EBT is valued for effectiveness in rehabilitation with lower perceived injury risk, while CT and PAP are associated with higher injury risk. RPT appears to occupy an intermediate position, being recognized for both effectiveness and moderate risk.

### 3.6. Capabilities Assessment in RT/AR

The higher operational and dynamic capabilities were analyzed to assess how specialists’ operational and dynamic skills—such as acquiring, integrating and applying knowledge, as well as adapting, absorbing and innovating—affect their effectiveness in rehabilitation and training (RT/AR).

#### 3.6.1. Operational Capabilities

ANOVA results for operational capabilities is presented in Table 6.

Specialists’ operational acquisition capabilities were assessed through their self-reported engagement with new trends, best practices and national networks. Overall, participants showed a moderate to high level of engagement: adopting new trends (M = 3.92, SD = 0.94), implementing best practices (M = 3.81, SD = 0.93) and participating in national networks (M = 4.10, SD = 0.95). RT+AR consistently reported higher scores across all three indicators (new trends: M = 4.38; SD = 0.71; best practices: M = 4.38; SD = 0.71; national networks: M = 4.88; SD = 0.34), suggesting greater operational capability in acquiring knowledge compared to RT or AR. ANOVA confirmed significant group differences for best practices (*p* = 0.002) and national networks (*p ≤* 0.001), while adoption of new trends did not differ significantly (*p* = 0.09).

For operational combination capabilities, related to knowledge integration, collaborative strategies and adoption of innovative ideas, overall ratings were moderately high: knowledge integration (M = 3.92, SD = 0.94), collaboration strategies (M = 3.60, SD = 1.10), and innovative ideas adoption (M = 4.10, SD = 0.77). Again, RT+AR scored higher across all indicators (knowledge integration: M = 4.63; SD = 0.61; strategies: M = 4.13; SD = 1.02; innovative ideas: M = 4.81; SD = 0.40), reflecting superior capacity for combining internal and external knowledge to optimize athlete recovery. Significant differences between groups were observed for all variables.

Analysis of operational acquisition and combination capabilities revealed notable differences between specialists with different roles. Regarding best practices, Tukey post hoc test revealed significant difference between RT+AR and RT (Mean difference = 1.12, *p* = 0.001). Games Howell post hoc test applied for national networks showed that RT+AR scored significantly higher than both RT (M difference = 1.56, *p* < 0.001) and AR (M difference = 0.75, *p* = 0.003). AR also scored higher than RT (M difference = 0.81, *p* = 0.02), suggesting greater engagement in national networks.

In terms of operational combination capabilities, RT+AR scored significantly higher than both RT (CC_knowledge_integration: M difference = 1.06, *p* = 0.005; CC_innovative_ideas_adoption: M difference = 1.12, *p* < 0.001) and AR (CC_knowledge_integration: M difference = 1.06, *p* = 0.003; CC_strategies: M difference = 1.00, *p* = 0.02; CC_innovative_ideas_adoption: M difference = 1.000, *p* = 0.001).

These results suggest that specialists combining rehabilitation and training responsibilities are consistently more engaged in professional development networks and demonstrate greater capacity for integrating knowledge and adopting innovative strategies compared to their single-domain counterparts.

#### 3.6.2. Higher-Order Dynamic Capabilities (DC)

ANOVA results for higher-order dynamic capabilities is presented in Table 7.

RT+AR consistently reported higher scores across all higher-order dynamic capabilities compared to RT and AR. For adaptive capability, RT+AR specialists indicated a greater ability to quickly identify change needs and adopt new strategies (M = 4.81, SD = 0.40) and to align with best practices (M = 4.81, SD = 0.54), with significant differences. For adaptive changes and absorptive capability, Games-Howell comparisons show that RT+AR reported significantly higher scores than RT for identifying change needs (M difference = 1.25, *p* = 0.001) and aligning with best practices (M difference = 1.25, *p ≤* 0.001). Differences between RT+AR and AR were also significant for aligning with best practices (M difference = 0.75, *p* = 0.03), while differences between RT and AR were not significant for these measures.

In terms of absorptive capability, RT+AR specialists scored highest for collaborations (M = 4.88, SD = 0.34) and training (M = 4.94, SD = 0.25). Games-Howell tests indicate that RT+AR scored higher than RT for strategic collaborations (M difference = 0.87, *p* < 0.001) and training (M difference = 0.62, *p* = 0.009), with AR comparisons showing smaller but still significant differences in training (M difference = 0.43, *p* = 0.04).

For innovative capability, RT+AR reported stronger development of new capabilities (M = 4.75, SD = 0.44) and adoption of innovations providing competitive advantages in rehabilitation (M = 4.75, SD = 0.57), with all differences statistically significant (*p* ≤ 0.001). Tukey HSD comparisons revealed that RT+AR significantly outperformed RT and AR in developing new capabilities (RT+AR vs. RT: M difference = 0.5, *p* = 0.05; RT+AR vs. AR: M difference = 0.81, *p ≤* 0.001) and implementing innovations (RT+AR vs. RT: M difference = 1.18, *p* < 0.001; RT+AR vs. AR: M difference = 0.75, *p* = 0.009).

Overall, these results indicate that RT+AR experience is associated with higher adaptive, absorptive and innovative capabilities in sports rehabilitation.

## 4. Discussion

The present study investigated the use and perceived effectiveness of advanced resistance training methods among RT, AR and RT+AR. The results provided substantial support for the proposed hypotheses.

H1 predicted that combined specialists would report more frequent use of advanced resistance training methods compared to single-domain specialists. In line with this, specialists combining resistance training and active recovery (RT+AR) reported more frequent use of advanced resistance training methods compared to single-domain specialists. This pattern was consistent across all analyzed methods—EBT-3/7, Cluster Sets, RPT and PAP—with statistically significant differences observed for each. These results indicate that specialists with combined expertise in rehabilitation and training engage more frequently and perceive greater effectiveness in advanced training methods, supporting H1.

Regarding H2, the results partially confirmed that perceptions of training methods’ effectiveness differed according to professional field. While EBT-3/7 was rated highly and similarly across all groups, significant differences emerged for CT, RPT and PAP. For CT, AR reported higher effectiveness in improving muscle strength than both RT and RT+AR. For RPT, differences were observed in perceived effects on workload and neuromuscular response. PAP showed significant differences in perceived enhancement of explosive power, with RT+AR rating it higher than both RT and AR. These findings suggest that perceptions of method effectiveness are influenced by professional specialization, partially validating H2.

H2a is not fully supported by the obtained results. While the EBT-3/7 method is generally perceived as effective for recovery across groups, the expected stronger perception among RT+AR specialists was not confirmed by the data, as the RT group actually rated post-workout recovery higher (M = 4.69) than the combined RT+AR group (M = 4.25).

H2b is partially supported. Cluster Training was generally perceived as effective for rehabilitation (91.1%), particularly in enhancing muscle strength, neuromuscular adaptation and recovery between sets. Overall, all groups gave positive ratings for strength improvement, recovery and neuromuscular adaptation, indicating that CT contributes meaningfully to rehabilitation outcomes, with differences between professional groups observed mainly for cluster set utilization and strength gains.

Most specialists reported using the RPT method occasionally, recognizing its role in increasing workload tolerance and enhancing training efficiency. RT+AR specialists showed significantly higher utilization compared to AR, while RT specialists rated the effect on increased workload significantly higher than both RT+AR and AR. Ratings for muscle tissue rehabilitation were consistently high across all groups (4.56 ± 0.586). Notably, RT+AR specialists reported significantly higher neuromuscular response values compared to RT, confirming the method’s efficiency in stimulating neuromuscular adaptations without compromising recovery. Neuromuscular control was rated similarly across groups. Overall, these findings validate H2c, indicating that RPT effectively enhances strength performance and neuromuscular response while supporting safe and progressive workload management in both rehabilitation and athletic contexts.

H2d is supported. For perceived effectiveness, RT+AR rated PAP highest for explosive power (4.87 ± 0.516), significantly more than both RT and AR, while reaction speed and neuromuscular coordination were highly rated across all groups with no significant differences. These findings indicate that PAP is perceived as particularly effective for enhancing explosive power and maintaining high neuromuscular coordination, supporting its application in both rehabilitation and athletic performance settings.

H3 predicted that methods rated as more effective for rehabilitation would be associated with lower perceived injury risk, while riskier methods would be less used in rehabilitation. This hypothesis was confirmed. EBT-3/7, rated as the most effective for rehabilitation, was associated with low perceived injury risk. In contrast, CT and PAP, although effective in specific performance domains, were perceived as carrying higher injury risk and were less frequently used for rehabilitation purposes. Specialists’ perceptions of injury risk appear to influence the selection of rehabilitation methods. RPT occupied an intermediate position, being recognized for both effectiveness and moderate risk. These findings highlight the trade-off between perceived efficacy and safety in method selection.

H4 stated that RT+AR would demonstrate higher operational and dynamic capabilities compared to single-domain specialists. The results fully support this hypothesis. Across all measures of operational capabilities—including engagement with new trends, adoption of best practices and participation in professional networks—RT+AR scored higher than RT or AR. Similarly, for higher-order dynamic capabilities such as adaptive, absorptive and innovative capacities, RT+AR demonstrated superior performance, with significant differences observed for all relevant variables. These findings indicate that combined-domain specialists not only employ advanced methods more effectively but also possess enhanced professional and dynamic competencies.

The results of the present study indicate that EBT-3/7 is perceived by specialists as highly effective for post-injury rehabilitation, particularly in optimizing muscle recovery and facilitating readaptation, consistent with prior research highlighting the method’s ability to harmonize volume, frequency and intensity while combining mechanical and metabolic stress for enhanced adaptation [25]. The incorporation of unilateral exercises, such as single-leg squats and single-arm rows, allows for correction of muscular imbalances and enhances functional recovery, highlighting the importance of targeted limb-specific training in rehabilitation protocols.

CT was also reported as effective in improving muscle strength and neuromuscular adaptation, consistent with prior evidence showing that short intra-set rest periods within cluster sets support performance maintenance without compromising movement velocity [27]. Cluster sets have also been shown to mitigate fatigue and reduce perceived exertion while maintaining or exceeding the training outcomes of traditional straight sets [28]. These characteristics make cluster sets particularly suitable for integration into unilateral resistance training sessions, providing a high-density stimulus while minimizing injury risk. Similarly, RPT was found to enhance muscular endurance and strength while balancing volume and intensity, providing a safe method for progressing workload without overtaxing recovering tissues.

PAP was identified as beneficial for neuromuscular coordination and explosive power, particularly in specialists with combined experience in rehabilitation and sports training. The present findings regarding the perceived benefits of PAP in rehabilitation contexts can be interpreted in light of recent neurophysiological evidence. Del Vecchio et al. demonstrated that motor unit synchronization arises from multiple independent sources of common synaptic input, underscoring the complexity of neuromuscular coordination [37]. This observation provides a mechanistic explanation for the potential effectiveness of PAP protocols, which aim to acutely enhance motor unit recruitment, firing rate and synchronization, thereby improving explosive power and neuromuscular efficiency [33,34]. In rehabilitation, where restoring motor coordination and functional performance is critical, the application of PAP-based strategies—such as combining high-force contractions with subsequent high-velocity tasks—may facilitate a faster and more effective return to play.

Overall, these findings suggest that an integrated rehabilitation strategy, combining EBT-3/7, CT, RPT and PAP with unilateral exercises, may optimize recovery outcomes by enhancing strength, neuromuscular control and functional adaptation. The progressive application of these methods, guided by pain, range of motion and movement quality, aligns with evidence-based principles and specialist perceptions, providing a comprehensive approach to post-injury rehabilitation.

**Figure 1:**
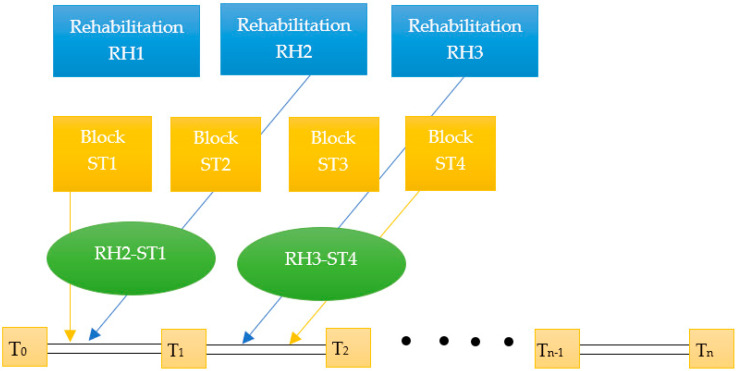
The concept of the rehabilitation strategy—periodization of the RH_i_-ST_j_ strategic mix.

Figure 1 presents the concept of the rehabilitation strategy—periodization of the RH_i_–ST_j_ strategic mix. In the first row, the rehabilitation strategies (RH1, RH2, …) are shown, while in the second row, the strength training blocks (ST1, ST2, …) are presented. These blocks can be combined in various personalized configurations (RH_i_–ST_j_), adapted to the subperiods (T_k_–T_k+1_) within the complete cycle for which the strategy was designed (T_0_–T_n_). This synergistic approach leverages the interactions between performance maintenance, accelerated recovery and the control of athletes’ confidence.

**Figure 2:**
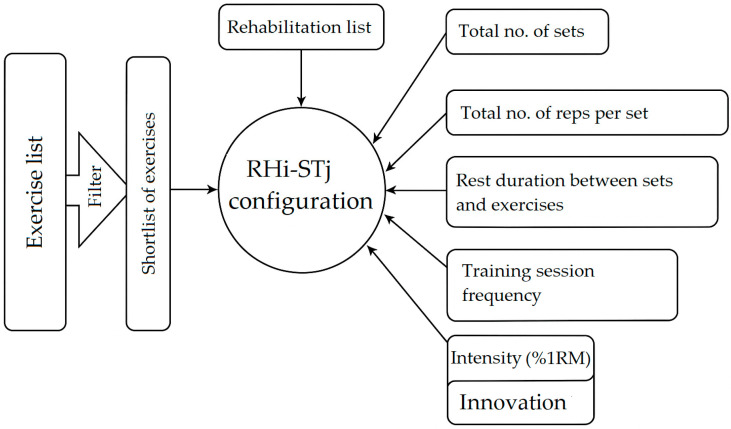
Configuring the RH_i_-ST_j_ strategy mix.

In Figure 2, the essential elements in configuring the strategy mix for a rehabilitation—strength training (RH_i_-ST_j_) module within the periodized program are highlighted. On the left branch, there is a list of exercises, followed by a filtering process to obtain a restricted, personalized list according to each case. At the top, there is a rehabilitation list developed by experts in this field in collaboration with strength training specialists.

On the right branch, the elements related to training volume are highlighted (total number of sets and total number of reps per set), along with factors related to rest duration between sets and exercises, training session frequency, intensity (expressed as %1RM) and innovative elements in the execution of exercises and sets. This aspect is essential in unilateral training dedicated to this type of rehabilitation.

All parameters are crucial in configuring the RH_i_-ST_j_ strategy in a personalized manner, adapted to particular cases. The proposed framework for designing efficient rehabilitation strategies offers elite athletes affected by seemingly minor injuries—but with a major impact on sports performance, such as tendinitis, tendinosis and tendinopathy—a new perspective that goes beyond in-depth understanding and modern pain monitoring capabilities. It opens opportunities for maintaining an acceptable level of performance and creates a robust foundation for rapid recovery. Building on this innovative framework for designing personalized rehabilitation strategies, the approach advances rehabilitation science beyond the classical stages (isometrics, progressive resistance, energy storage and release, return to sport/activities), introducing a new focus on the duration and quality of recovery in elite athletes. In this context, strength training blocks are more precisely oriented toward the intensity of exercises performed on the unaffected side and the intelligent manipulation of force–time profiles.

The present study has several limitations that should be acknowledged. First, the findings are based primarily on subjective perceptions and self-reported evaluations from specialists, which may introduce response bias and limit the objectivity of the results. The absence of physiological or performance-based outcome measures restricts the ability to validate these perceptions through empirical data. Second, the study captures a snapshot of expert opinion at a single point in time, without tracking the actual outcomes of implementing advanced resistance training methods in rehabilitation programs. Longitudinal or experimental research is needed to evaluate the sustained impact of these approaches on recovery and performance. Third, potential professional bias should be considered. Specialists practicing in both rehabilitation and training reported higher perceived effectiveness and utilization of all methods, which may reflect their broader exposure and integrative expertise but also the influence of professional confidence or familiarity.

Future research will investigate the longitudinal effects of integrating EBT, CT, RPT and PAP in rehabilitation protocols on objective performance and recovery outcomes. Additionally, studies could explore larger and more diverse samples of specialists and athletes, consider sport-specific adaptations and evaluate the method-specific benefits—particularly those related to neuromuscular coordination and injury prevention. Furthermore, the development of standardized implementation guidelines based on specialist expertise and evidence-based practice could further optimize rehabilitation outcomes.

## 5. Conclusions

The present study demonstrates that, according to the specialists’ opinions, advanced resistance training methods are perceived as effective tools in post-injury rehabilitation of high-performance athletes, with method selection influenced by professional specialization and perceived injury risk. Among these methods, EBT-3/7 was consistently rated as highly effective and safe, supporting muscle recovery and readaptation. CT and PAP were considered to provide performance-enhancing benefits, particularly in strength, neuromuscular adaptation and explosive power, but were associated with higher perceived injury risk, requiring careful implementation. RPT was considered a balanced approach, enhancing muscular endurance and strength while moderating risk.

Specialists with combined expertise in rehabilitation and training reported higher utilization and perceived effectiveness across all methods compared to single-domain specialists. They also demonstrated superior operational and dynamic capabilities, including adaptive, absorptive and innovative capacities, suggesting that integrated professional experience contributes to more effective implementation of advanced training strategies.

Overall, the findings indicated that advanced resistance training methods are perceived by specialists as effective and safe for accelerating post-injury recovery and readaptation in elite athletes, with combined rehabilitation and training expertise enhancing method utilization, perceived effectiveness and the implementation of personalized, performance-oriented rehabilitation strategies.

## Figures and Tables

**Table 1:** Main intensification strategies.

Strategy	Description	Example
Progressive overload	Gradually increasing the resistance or complexity of the exercises to continuously challenge the neuromuscular system.	Late-stage ACL-reconstructed athletes performing eccentric-oriented strength training using flywheel devices showed greater neuromuscular demand compared with traditional strength training over six weeks, illustrating the application of progressive overload in rehabilitation [14].
Volume progression	Increasing the total number of repetitions over time to enhance training stimulus and adaptation.	In eccentric versus traditional ACL rehabilitation, the eccentric group performed 64 to 120 repetitions spread over four exercises across the rehab period, representing a progressive increase in training volume compared with standard protocols [14].
Reducing reps in reserve (RIR)	Lowering the number of repetitions left before failure to push athletes closer to their maximum capacity.	Although few rehabilitation studies explicitly manipulate repetitions in reserve due to safety concerns, some late-phase eccentric-oriented exercises, such as flywheel Bulgarian squats, are performed near maximal effort, suggesting that low RIR can be applied safely when the athlete’s tissues and functional tests allow [14].
Modification of repetition style	Altering tempo, range of motion or contraction type (e.g., eccentric, isometric holds) to vary the stimulus and optimize muscle engagement.	Comparing eccentric, plyometric or combined eccentric + plyometric training, plyometrics involve stretch-shortening cycle contractions and rapid eccentric-to-concentric transitions, highlighting variation in contraction style [15].

**Table 2:** ANOVA for EBT-3/7.

Variable	Category	N	M	SD	CI95% Lower	CI95% Upper	*p*	Levene Sig.
EBT3_7_utilization	RT	16	2.13	0.71	1.74	2.51	0.03	0.44
	AR	16	2.00	0.63	1.66	2.34		
	RT+AR	16	2.56	0.51	2.29	2.84		
	Total	48	2.23	0.66	2.04	2.42		
EBT3_7_effectiveness	RT	16	4.75	0.57	4.44	5.06	0.56	0.17
	AR	16	4.56	0.81	4.13	5.00		
	RT+AR	16	4.5	0.63	4.16	4.84		
	Total	48	4.6	0.67	4.41	4.80		
EBT3_7_postworkout_recovery	RT	16	4.69	0.60	4.37	5.01	0.032	≤0.001
	AR	16	3.81	1.10	3.22	4.40		
	RT+AR	16	4.25	0.93	3.75	4.75		
	Total	48	4.25	0.95	3.97	4.53		
EBT3_7_muscular_performance_ improvement	RT	16	4.56	0.51	4.29	4.84	0.61	0.03
	AR	16	4.56	0.81	4.13	5.00		
	RT+AR	16	4.75	0.44	4.51	4.99		
	Total	48	4.63	0.60	4.45	4.01		
EBT3_7_injuryrisk_reduction	RT	16	4.69	0.47	4.43	4.94	0.38	0.05
	AR	16	4.50	0.63	4.16	4.84		
	RT+AR	16	4.53	0.44	4.51	4.99		
	Total	48	4.65	0.60	4.49	4.80		
EBT3_7_readaptation	RT	16	4.56	0.51	4.29	4.84	0.21	0.003
	AR	16	4.50	0.64	4.16	4.84		
	RT+AR	16	4.81	0.41	4.60	5.03		
	Total	48	4.63	0.53	4.47	4.78		

N—number of respondents. M—average. SD—standard deviation. CI—interval of confidence. *p*—significant level of probability (ANOVA). Levene Sig.—Levene test level of significance. RT—sports rehabilitation specialists. AR—sports training specialists. RT+AR - both sports rehabilitation and sports training specialists.

**Table 3:** ANOVA for CT.

Variable	Category	N	M	SD	CI95% Lower	CI95% Upper	*p*	Levene Sig.
Cluster sets_utilization	RT	16	1.19	0.40	0.97	1.40	0.01	0.17
	AR	16	0.88	0.61	0.55	1.20		
	RT+AR	16	1.44	0.51	1.16	1.71		
	Total	48	1.17	0.55	1.00	1.33		
Drop Sets_utilization	RT	16	1.31	0.47	1.06	1.57	0.13	0.36
	AR	16	1.06	0.57	0.76	1.37		
	RT+AR	16	1.44	0.51	1.16	1.71		
	Total	48	1.27	0.53	1.12	1.43		
CT_effective_muscle_strenghth	RT	16	4.19	0.83	3.74	4.63	0.02	0.003
	AR	16	4.81	0.40	4.60	5.03		
	RT+AR	16	4.31	0.60	3.99	4.63		
	Total	48	4.44	0.68	4.24	4.64		
CT_Strenght_improvement	RT	16	4.63	0.50	4.36	4.89	0.27	0.006
	AR	16	4.88	0.34	4.69	5.06		
	RT+AR	16	4.75	0.44	4.51	4.99		
	Total	48	4.75	0.43	4.62	4.88		
CT_recovery_between_sets	RT	16	4.50	0.51	4.22	4.878	0.18	0.01
	AR	16	4.38	0.71	3.99	4.76		
	RT+AR	16	4.75	0.44	4.51	4.99		
	Total	48	4.54	0.58	4.37	4.71		
CT_neuromuscular_adaptation	RT	16	4.56	0.51	4.29	4.84	0.052	≤0.001
	AR	16	4.69	0.47	4.43	4.94		
	RT+AR	16	4.94	0.25	4.80	5.07		
	Total	48	4.73	0.44	4.60	4.86		

N—number of respondents. M—average. SD—standard deviation. CI—interval of confidence. *p*—significant level of probability (ANOVA). Levene Sig.—Levene test level of significance. RT—sports rehabilitation specialists. AR—sports training specialists. RT+AR—both sports rehabilitation and sports training specialists.

**Table 4:** ANOVA for RPT.

Variable	Category	N	M	SD	CI95% Lower	CI95% Upper	*p*	Levene Sig.
RPT_utilization	RT	16	1.25	0.44	1.01	1.52	0.03	0.04
	AR	16	0.94	0.44	0.68	1.19		
	RT+AR	16	1.38	0.50	1.12	1.68		
	Total	48	1.19	0.49	1.05	1.35		
RPT_increased_workload	RT	16	4.31	0.60	4.12	4.68	0.02	0.05
	AR	16	3.50	1.03	2.88	4.05		
	RT+AR	16	3.50	1.09	2.82	3.98		
	Total	48	3.77	0.99	3.45	4.06		
RPT_muscle_tissue_ rehabilitation	RT	16	4.56	0.51	4.25	4.82	0.31	0.01
	AR	16	4.44	0.72	3.99	4.81		
	RT+AR	16	4.75	0.44	4.48	4.99		
	Total	48	4.58	0.57	4.38	4.73		
RPT_neuromuscular_ response	RT	16	3.81	1.16	3.13	4.47	0.002	≤0.001
	AR	16	4.44	0.62	4.25	4.82		
	RT+AR	16	4.88	0.34	4.67	5.06		
	Total	48	4.38	0.89	4.13	4.67		
RPT_neuromuscular_ control	RT	16	4.31	0.87	3.78	4.76	0.1	0.001
	AR	16	4.56	0.51	4.32	4.88		
	RT+AR	16	4.81	0.54	4.49	5.11		
	Total	48	4.56	0.68	4.35	4.76		

N—number of respondents. M—average. SD—standard deviation. CI—interval of confidence. *p*—significant level of probability (ANOVA). Levene Sig.—Levene test level of significance. RT—sports rehabilitation specialists. AR—sports training specialists. RT+AR—both sports rehabilitation and sports training specialists.

**Table 5:** ANOVA for PAP.

Variable	Category	N	M	SD	CI95% Lower	CI95% Upper	*p*	Levene Sig.
PAP_utilization	RT	16	0.56	0.62	0.23	0.90	≤0.001	0.66
	AR	16	1.38	0.61	1.05	1.70		
	RT+AR	16	1.50	0.51	1.22	1.78		
	Total	48	1.15	0.71	0.94	1.35		
PAP_explosive_power	RT	16	4.25	0.77	3.84	4.65	0.004	0.02
	AR	16	3.81	1.16	3.19	4.43		
	RT+AR	16	4.88	0.5	4.61	5.14		
	Total	48	4.31	0.94	4.04	4.59		
PAP_reaction_speed	RT	16	4.38	1.08	3.80	4.95	0.14	0.008
	AR	16	4.06	1.43	3.30	4.83		
	RT+AR	16	4.81	0.40	4.60	5.03		
	Total	48	4.42	1.08	4.1	4.73		
PAP_neuromuscular_ coordination	RT	16	4.44	0.89	3.96	4.91	0.03	≤0.001
	AR	16	4.81	0.54	4.52	5.10		
	RT+AR	16	5.00	0	5.00	5.00		
	Total	48	4.75	0.63	4.57	4.93		

N—number of respondents. M—average. SD—standard deviation. CI—interval of confidence. *p*—significant level of probability (ANOVA). Levene Sig.—Levene test level of significance. RT—sports rehabilitation specialists. AR—sports training specialists. RT+AR—both sports rehabilitation and sports training specialists.

**Table 6:** ANOVA for operational capabilities.

Variable	Category	N	M	SD	CI95% Lower	CI95% Upper	*p*	Levene Sig.
AC_adopt_new_trends	RT	16	3.69	0.94	3.18	4.19	0.09	0.05
	AR	16	3.69	1.25	3.02	4.35		
	RT+AR	16	4.38	0.71	3.99	4.76		
	Total	48	3.92	1.02	3.62	4.22		
AC_best_practices	RT	16	3.25	0.85	2.79	3.71	0.002	0.38
	AR	16	3.81	0.91	3.33	4.30		
	RT+AR	16	4.38	0.71	3.99	4.76		
	Total	48	3.81	0.93	3.54	4.08		
AC_national_networks	RT	16	3.31	0.94	2.81	3.82	≤0.001	0.003
	AR	16	4.13	0.71	3.74	4.51		
	RT+AR	16	4.88	0.34	4.69	5.06		
	Total	48	4.10	0.95	3.83	4.38		
CC_knowledge_integration	RT	16	3.56	1.03	3.01	4.11	≤0.001	0.04
	AR	16	3.56	0.72	3.17	3.95		
	RT+AR	16	4.63	0.61	4.30	4.95		
	Total	48	3.92	0.94	3.64	4.19		
CC_strategies	RT	16	3.56	0.89	3.09	4.04	0.03	0.45
	AR	16	3.13	1.20	2.48	3.77		
	RT+AR	16	4.13	1.02	3.58	4.7		
	Total	48	3.60	1.10	3.28	3.93		
CC_innovative_ ideas_adoption	RT	16	3.69	0.47	3.43	3.94	≤0.001	0.03
	AR	16	3.81	0.83	3.37	4.26		
	RT+AR	16	4.81	0.40	4.60	5.03		
	Total	48	4.10	0.77	3.88	4.33		

N—number of respondents. M—average. SD—standard deviation. CI—interval of confidence. *p*—significant level of probability (ANOVA). Levene Sig.—Levene test level of significance. RT—sports rehabilitation specialists. AR—sports training specialists. RT+AR—both sports rehabilitation and sports training specialists.

**Table 7:** ANOVA for higher-order dynamic capabilities.

Variable	Category	N	M	SD	CI95% Lower	CI95% Upper	*p*	Levene Sig.
Adaptive_changes_needs	RT	16	3.56	1.03	3.01	4.11	≤0.001	0.001
	AR	16	4.38	0.80	3.95	4.80		
	RT+AR	16	4.81	0.40	4.60	5.03		
	Total	48	4.25	0.93	3.98	4.52		
good_practices_alignment	RT	16	3.56	0.96	3.05	4.08	≤0.001	0.004
	AR	16	4.06	0.92	3.57	4.56		
	RT+AR	16	4.81	0.54	4.52	5.10		
	Total	48	4.15	0.96	3.86	4.43		
DC_AbC_collaborations	RT	16	4.00	0.51	3.72	4.28	≤0.001	0.008
	AR	16	4.44	0.62	4.10	4.77		
	RT+AR	16	4.88	0.34	4.69	5.06		
	Total	48	4.44	0.61	4.26	4.62		
DC_AbC_training	RT	16	4.31	0.70	3.94	4.69	0.01	≤0.001
	AR	16	4.50	0.63	4.16	4.84		
	RT+AR	16	4.94	0.25	4.80	5.07		
	Total	48	4.58	0.61	4.41	4.76		
IC_newCapabilities	RT	16	4.25	0.68	3.89	4.61	0.001	0.19
	AR	16	3.94	0.57	3.63	4.24		
	RT+AR	16	4.75	0.44	4.51	4.99		
	Total	48	4.31	0.65	4.12	4.50		
IC_Innovations	RT	16	3.56	0.89	3.09	4.04	≤0.001	0.10
	AR	16	4.00	0.73	3.61	4.39		
	RT+AR	16	4.75	0.57	4.44	5.06		
	Total	48	4.10	0.88	3.85	4.36		

N—number of respondents. M—average. SD—standard deviation. CI—interval of confidence. *p*—significant level of probability (ANOVA). Levene Sig.—Levene test level of significance. RT—sports rehabilitation specialists. AR—sports training specialists. RT+AR—both sports rehabilitation and sports training specialists.

## Data Availability

The data used to support the findings of the current study are available from the corresponding authors upon request.
